# The prognostic impacts of *TEA domain (TEAD)* transcription factor polymorphisms in Chinese hepatocellular carcinoma patients

**DOI:** 10.18632/oncotarget.19310

**Published:** 2017-07-17

**Authors:** Haiyan Xia, Juan Wen, Weiyong Zhao, Dongying Gu, Zhibin Hu, Jinfei Chen, Zhi Xu

**Affiliations:** ^1^ Department of Oncology, Nanjing First Hospital, Nanjing Medical University, Nanjing, China; ^2^ Nanjing Maternity and Child Health Care Institute, Nanjing Maternity and Child Health Care Hospital, Obstetrics and Gynecology Hospital Affiliated to Nanjing Medical University, Nanjing, China; ^3^ Department of Epidemiology and Biostatistics, Jiangsu Key Laboratory of Cancer Biomarkers, Prevention and Treatment, Cancer Center, School of Public Health, Nanjing Medical University, Nanjing, China; ^4^ Department of Radiation Oncology, The Second Affiliated Hospital, Nanjing Medical University, Nanjing, China; ^5^ State Key Laboratory of Reproductive Medicine, Nanjing Medical University, Nanjing, China

**Keywords:** TEA domain family member, single nucleotide polymorphism, hepatocellular carcinoma, prognosis

## Abstract

TEA domain (TEAD) transcription factors play an important role in hepatocellular carcinoma (HCC) development and progression by regulating the expression of a number of genes. However, the association of their genetic variations with HCC prognosis remains elusive. Seven potentially functional single nucleotide polymorphisms in *TEAD1*-*4* (rs2304733, rs10831923, rs12104362, rs3745305, rs11756089, rs2076173, rs7135838) were genotyped from 331 hepatitis B virus positive HCC patients using the Sequenom MassARRAY iPLEX platform. The *TEAD3* rs2076173 C allele and rs11756089 T allele were identified as protective alleles as they were significantly associated with longer median overall survival time (MST). The T allele of rs2076173 was significantly associated with HCC survival independent of age, gender, smoking and drinking status, BCLC stage, and chemotherapy or TACE status (HR = 0.73, 95% CI = 0.56-0.93, *P* = 0.012). This protective effect was more prominent for patients who were non-drinkers (*P* for multiplicative interaction = 0.002). Patients had more than one of these protective alleles had significant longer MST of 19.25 months than those had none (MST=12.85 months, adjusted HR = 0.56, 95% CI = 0.33-0.95, *P*=0.030), especially for those non-drinkers (adjusted HR = 0.48, 95% CI = 0.32-0.74, *P* = 0.001). These findings suggested that rs2076173 and rs11756089 in *TEAD3* gene could serve as genetic markers for favorable survival in the Chinese HCC patients.

## INTRODUCTION

Hepatocellular carcinoma (HCC) is the fourth commonly diagnosed cancer and the third leading cause of cancer death in China [1]. Due to the asymptomatic nature, HCC are often diagnosed at advanced stage with poor prognosis. Combined modalities such as surgery, radiation, chemotherapy and molecular targeted therapy were showed to provide limited or marginal therapeutic benefits and always associated with unpleasant side effects. There remains an urgent need to identify molecular markers that can identify high-risk patients for more appropriate cancer treatment that gives a better clinical outcome.

The transcriptional enhancer activator domain (TEAD) family is a group of transcriptional factors, participating in the development of various tumors, such as liver [2], gastric [3] and ovarian cancers [4]. Four family members (TEAD1, TEAD2, TEAD3 and TEAD4) shared in the highly conserved TEA DNA binding domain and required transcriptional coactivators for transcription activation [5]. With the help of coactivators, quite a few genes relevant to tumorigenesis are regulated by TEADs, including *connective tissue growth factor* (*CTGF*) [6], *AXL tyrosine kinase receptor* (*AXL*) [7], *cyclin D1* (*CCND1*) and *Forkhead box protein M1* (*FOXM1*) [8]. In HCC, TEAD-Yes-associated protein (YAP) complex is the downstream regulator of the Hippo tumor suppressor pathway [9]. Dysregulated Hippo signaling pathway will lead to hypophosphorylation of YAP and resulting in translocation of YAP into the nucleus to form the complex with TEADs. The four TEAD family members have similar affinity for YAP. The YAP-TEAD complex subsequently would induce a number of gene expressions involving anti-apoptosis, proliferation and “stemness” phenotype [9–11]. Disrupting YAP-TEAD complex was demonstrated to impede HCC tumorigenesis without interrupting normal liver homeostasis [12]. Other evidences showed that downregulation of TEAD1/3/4 were able to abolish YAP-induced oncogenic transformation including cell proliferation, anchorage-independent growth and epithelial-mesenchymal transition (EMT)suggesting that TEAD is essential for the function of YAP in HCC development and progression [9]. Genetic variations of *TEADs* such as*TEAD1* rs7944031 AG/GG and *TEAD4* rs1990330 CA/AA genotypes, were significantly associated with poor survival time in melanoma [13]. An alternative splicing form of *TEAD4* which lacking the N-terminal DNA-binding domain but maintaining the YAP interaction domain was found under-expressed in various cancer cells. Restoring this TEAD4 isoform could inhibit tumor growth through repressing YAP signaling [14].

Nevertheless, the clinical significance of *TEADs* genetic variations in HCC has not been known. Considering the essential role of TEADs in HCC development and progression, we performed single nucleotide polymorphisms (SNPs) genotyping on seven potentially functional variants of *TEADs* in 331 HCC patients to investigate whether they could serve as biomarkers for better clinical management.

## RESULTS

### Association of *TEADs* polymorphisms with overall survival

The demographic characteristics and clinical information of the 331 HCC patients were described previously [15]. There were 258 of patients who died from HCC, and 2 died from other causes during the follow-up to 60.7 months. Median age of the patients was 53 years and the median survival time (MST) was 14.5 months. Of the 331 patients, 284 patients (85.8%) were male and 47 (14.2%) were female; 211 (63.7%) patients were defined as smokers and 204 (61.6%) patients were drinkers; 304 (91.8%) patients were at BCLC stage B and 27 (8.2%) of them at BCLC stage C; 240 (72.5%) received either the chemotherapy or TACE therapy. Notably, the drinkers had a higher risk of death (HR = 1.43, 95%CI = 1.11-1.84, *P* = 0.006) than non-drinkers, whereas patients with chemotherapy or TACE had a 61% significant risk reduction of death (HR = 0.39, 95%CI = 0.29-0.51, *P* < 0.001).

**Figure 1:**
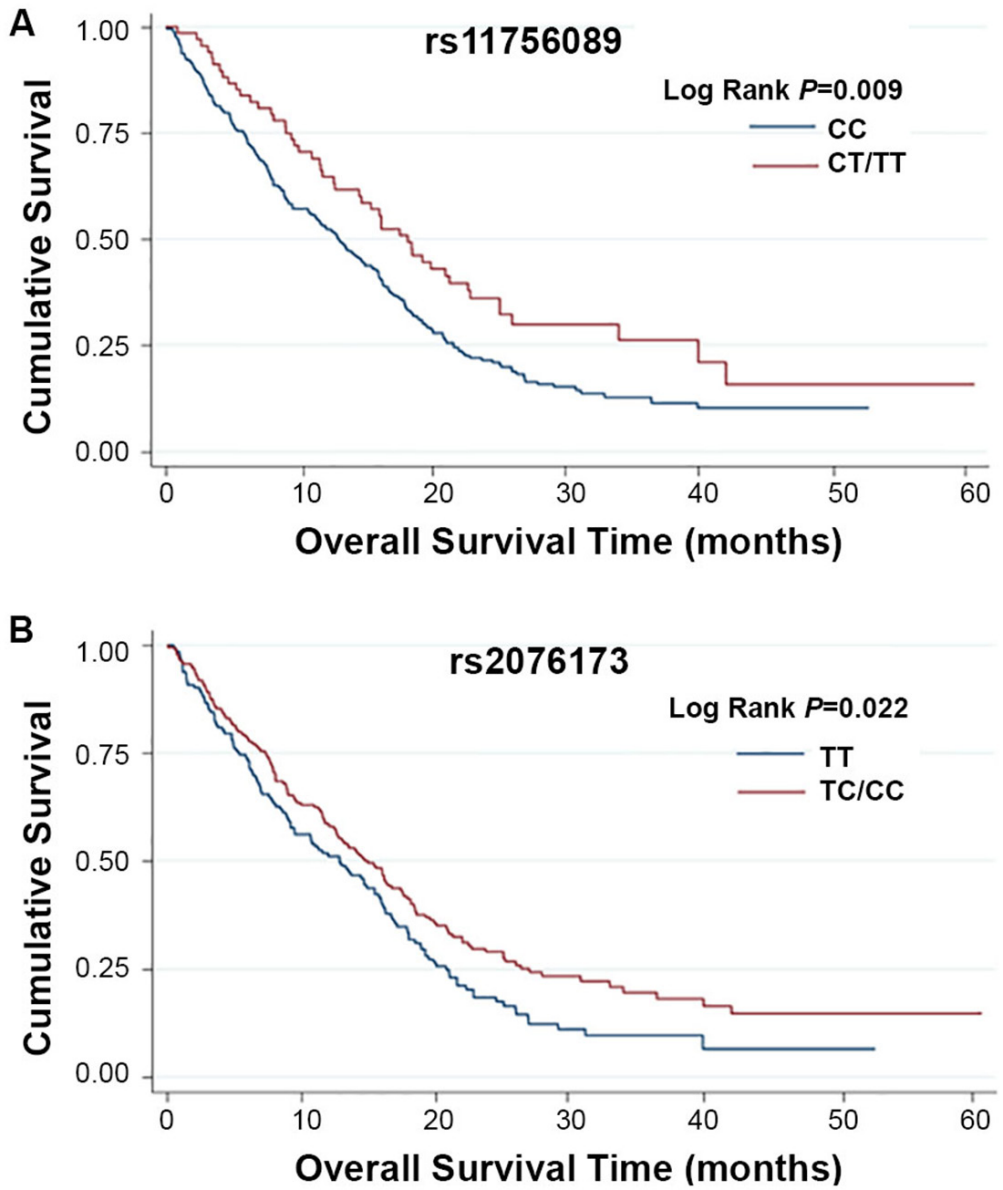
Kaplan-Meier plots of survival by *TEAD3* rs11756089 and rs2076173 genotypes in HCC patients’ survival **(A)** *TEAD3* rs11756089 genotypes and HCC survival (log-rank *P* = 0.009 for CT/TT vs. CC) in a dominant model. **(B)** *TEAD3* rs2076173 genotypes and HCC survival (log-rank *P* = 0.022 for TC/CC vs. TT) in a dominant model.

Kaplan-Meier and log-rank tests were used to examine the associations between the SNPs and HCC survival by using different genetic models. All *P*-values of Hardy-Weinberg equilibrium were greater than 0.05. None of the SNPs tested for TEAD1/2/4 were found to have significant association with the overall survival time. Two SNPs of *TEAD3*, rs11756089 and rs2076173 polymorphisms were significantly correlated with the HCC specific overall survival time using the dominant model (log-rank test: *P* = 0.009 and *P* = 0.022, respectively) and the additive model (log-rank test: *P* = 0.024 and *P* = 0.039, respectively) (Table 1 and Figure 1). As shown in Table 2, compared to the patients carrying rs11756089 CC genotype, those with CT/TT genotypes had a longer MST of 18.14 months, although not reaching statistical significance after adjusted for age, gender, smoking and drinking status, BCLC stage, and chemotherapy or TACE status (adjusted HR = 0.74, 95% CI = 0.53-1.03, *P* = 0.075). For rs2076173, TC/CC genotypes was shown to result in a significant improvement in overall survival time of 14.95 months (adjusted HR = 0.73, 95% CI = 0.56-0.93, *P* = 0.012) when compared to TT genotype with MST of 12.85 months. Since both of the SNPs are located within *TEAD3* gene, the combined effects of the protective alleles (T allele of rs11756089 and C allele of rs2076173) on HCC survival were examined (Table 2). Patients carrying increasing number of these protective alleles tends to have better survival outcomes (*P* for trend = 0.011). Patients with 1-4 protective alleles had a 27% significant risk reduction of death (95% CI = 0.57-0.94, *P* = 0.014), compared to patients with wide-type (WT) homozygotes of the two SNPs. Similarly, patients with 3-4 protective alleles had a significantly longer MST of 19.25 months than those had none (MST = 12.85 months, adjusted HR = 0.56, 95% CI = 0.33-0.95, *P* = 0.030).

**Table 1:** Genotyping results with HCC patients' survival

SNP	Base change ^a^	Gene	Location	Genotyping Rate	MAF ^b^	Log-rank *P*	
						Dominant model	Additive model
rs2304733	T>C	TEAD1	11p15.2	96.98%	0.132	0.137	0.180
rs10831923	T>A	TEAD1	11p15.2	96.07%	0.414	0.900	0.966
rs12104362	T>C	TEAD2	19q13.3	97.89%	0.500	0.537	0.158
rs3745305	C>T	TEAD2	19q13.3	97.58%	0.067	0.100	0.100
rs11756089	C>T	TEAD3	6p21.31	96.37%	0.114	0.009	0.024
rs2076173	T>C	TEAD3	6p21.31	96.68%	0.354	0.022	0.039
rs7135838	C>G	TEAD4	12p13.33	96.68%	0.159	0.130	0.241

HCC, hepatocellular carcinoma; SNP, single nucleotide polymorphism; MAF, minor allele frequency.

^a^ major > minor allele.

^b^ MAF in Patients.

**Table 2:** Polymorphisms and HCC Patients' Survival

Genotype	Patients	Deaths	MST (months)	Crude HR (95% CI)	Adjusted HR (95% CI) ^a^	*P*^a^
rs11756089						
CC	257	209	12.98	1.00	1.00	
CT	62	44	16.16	0.70 (0.50-0.97)	0.75 (0.53-1.05)	0.097
TT	6	4	22.87	0.42 (0.15-1.12)	0.66 (0.24-1.80)	0.418
Additive model				0.68 (0.52-0.90)	0.77 (0.57-1.03)	0.078
Dominant model				0.66 (0.48-0.91)	0.74 (0.53-1.03)	0.075
rs2076173						
TT	142	120	12.85	1.00	1.00	
TC	137	108	14.13	0.79 (0.61-1.03)	0.76 (0.58-0.99)	0.041
CC	47	30	16.26	0.63 (0.42-0.93)	0.63 (0.42-0.94)	0.025
Additive model				0.79 (0.66-0.95)	0.78 (0.65-0.94)	0.009
Dominant model				0.75 (0.59-0.96)	0.73 (0.56-0.93)	0.012
Combined genotypes (rs11756089-T and rs2076173-C)						
0	140	118	12.85	1.00	1.00	
1	97	78	13.14	0.86 (0.64-1.14)	0.76 (0.57-1.02)	0.071
2	61	44	16.03	0.68 (0.48-0.97)	0.74 (0.52-1.05)	0.095
3-4	26	17	19.25	0.57 (0.34-0.95)	0.56 (0.33-0.95)	0.030
Trend				*P* ^b^ = 0.005	*P* ^b^ = 0.011	
0	140	118	12.85	1.00	1.00	
1-4	184	139	14.95	0.75 (0.59-0.96)	0.73 (0.57-0.94)	0.014

HCC, hepatocellular carcinoma; MST, median survival time; HR, hazard ratio; CI, confidence intervals.

^a^ Adjusted for age, gender, smoking and drinking status, BCLC stage, and chemotherapy or TACE status.

^b^ *P* value of Cochran-Armitage’s trend test.

### Stepwise cox regression analysis on HCC survival

We then performed stepwise Cox proportional hazard analysis to estimate the effects of demographic characteristics, clinical features, *TEAD3* rs11756089 and rs2076173 on HCC survival. As shown in Table 3, four variables (chemotherapy or TACE status, age, *TEAD3* rs2076173 and drinking status) were selected into the final regression model. Furthermore, when gender, smoking status and BCLC stage were included in the final model, the *TEAD3* rs2076173 still remained as an independent protective factor for HCC survival (HR = 0.69, 95%CI = 0.54-0.89, *P* = 0.005).

**Table 3:** Multivariable cox regression analysis on HCC patients’ survival

Variables	β ^a^	SE ^b^	HR	95% CI	*P*
*Stepwise regression analysis*					
Chemotherapy or TACE (yes *vs.* none)	-1.1340	0.1524	0.32	0.24-0.43	<0.001
Age (>53 *vs.* <=53)	-0.4467	0.1366	0.64	0.49-0.84	0.001
rs2076173 (TC/CC *vs.*TT)	-0.3580	0.1293	0.70	0.54-0.90	0.006
Drinking status (yes *vs.* no)	0.3112	0.1321	1.37	1.05-1.77	0.018
*Final regression model*					
Chemotherapy or TACE (yes *vs.* none)	-1.1419	0.1523	0.32	0.24-0.43	<0.001
Age (>53 *vs.* <=53)	-0.4555	0.1363	0.63	0.49-0.83	0.001
rs2076173 (TC/CC *vs.*TT)	-0.3644	0.1289	0.69	0.54-0.89	0.005
Drinking status (yes *vs.* no)	0.3074	0.1320	1.36	1.05-1.76	0.020

HCC, hepatocellular carcinoma; HR, hazard ratio; CI, confidence intervals; TACE, transcatheter hepatic arterial chemoembolization.

^a^ β is the estimated parameter of the regression model.

^b^ SE is the standard error of the regression model.

### *TEAD3* polymorphisms associated with survival in HCC patients’ subtypes

**Figure 2:**
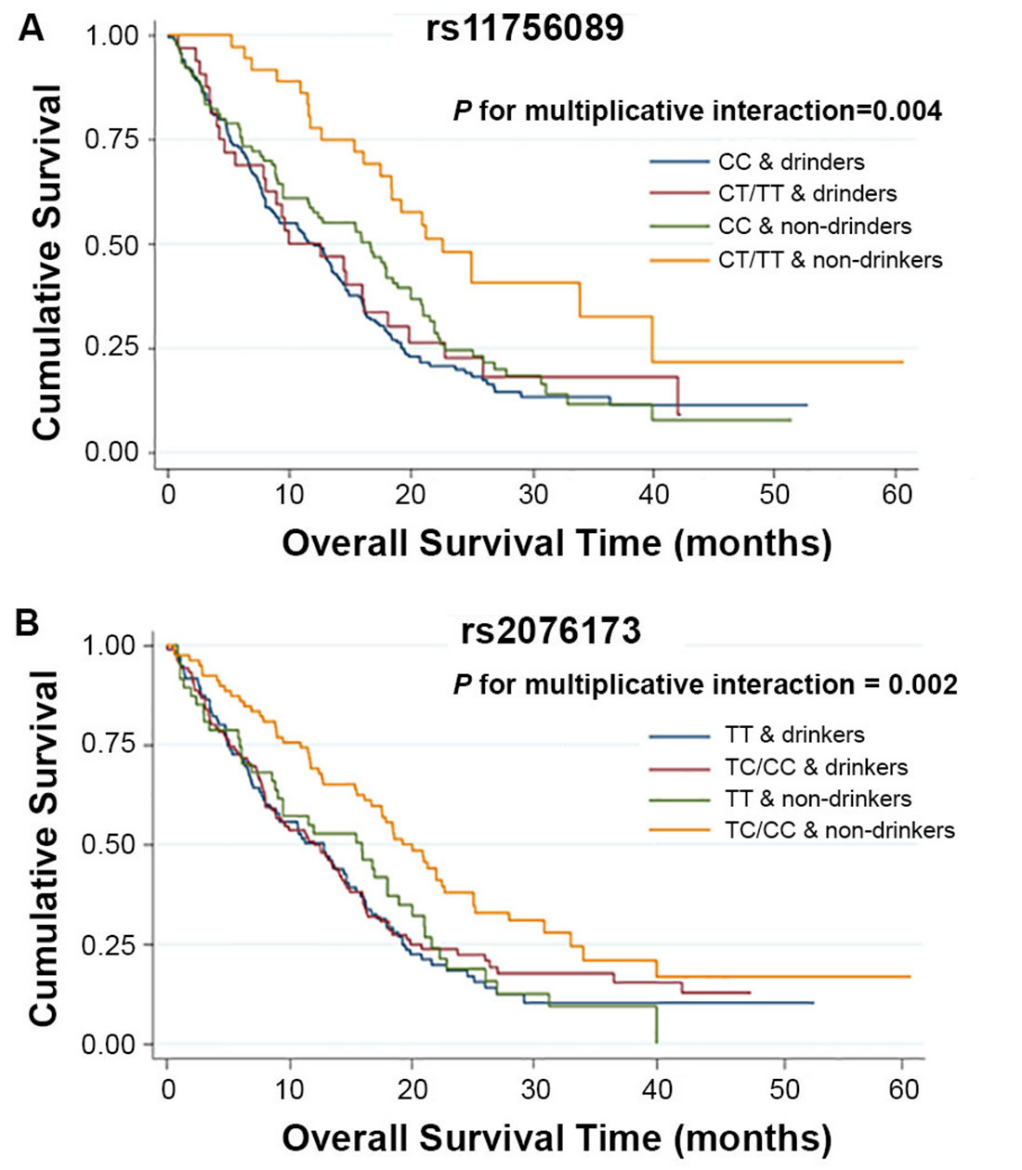
Kaplan-Meier plots of survival by *TEAD3* rs11756089 and rs2076173 genotypes in HCC patients’ survival **(A)** Kaplan-Meier plots of survival by the combination of rs11756089 genotypes and drinking status in HCC-specific survival (*P* for multiplicative interaction = 0.004). **(B)** Kaplan-Meier plots of survival by the combination of rs2076173 genotypes and drinking status in HCC-specific survival (*P* for multiplicative interaction = 0.002).

**Figure 3:**
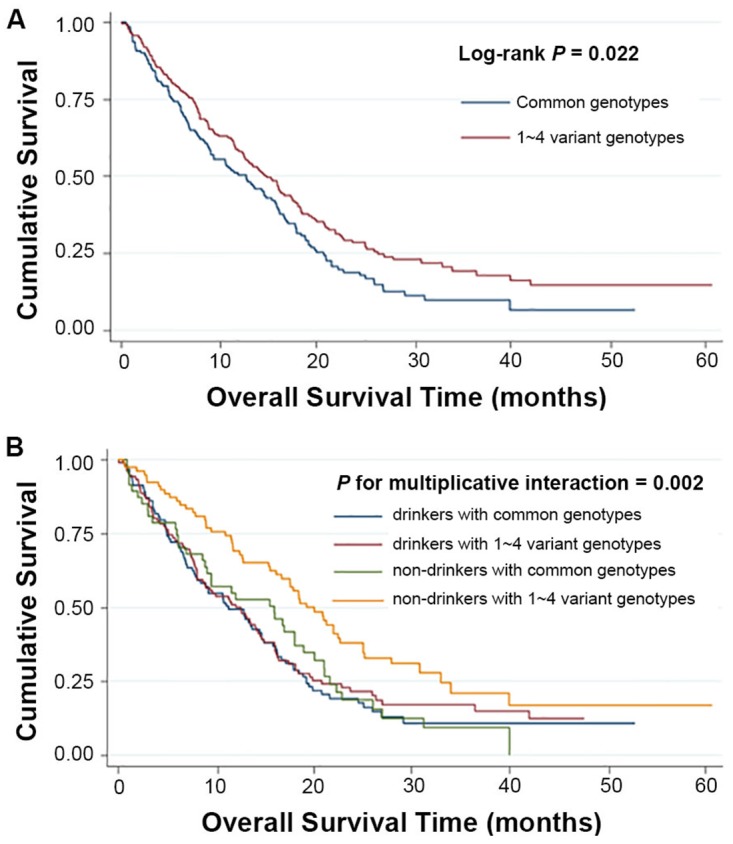
Kaplan-Meier plots of survival by combined genotypes (rs11756089-T and rs2076173-C) in HCC patients’ survival **(A)** combined genotypes and HCC survival (Log-rank *P* = 0.022 for 1∼4 variant genotypes vs. common genotypes). **(B)** Kaplan-Meier plots of survival by the combination of combined genotypes and drinking status in HCC-specific survival (*P* for multiplicative interaction = 0.002).

To better assess the correlations of the two polymorphisms of *TEAD3* and HCC survival, stratified analysis was performed on age, gender, smoking and drinking status, BCLC stage, and chemotherapy/TACE status. As shown in Supplementary Tables 2 and 3, the protective effects of the variant genotypes of rs11756089 and rs2076173 were more prominent in non-drinkers than drinkers (heterogeneity test: *P* = 0.002 for rs11756089; *P* = 0.031 for rs2076173). Similarly, the combined protective effect of the variant alleles of rs11756089 and rs2076173 was also more prominent in non-drinkers (adjusted HR = 0.50, 95% CI = 0.33-0.77) than drinkers (adjusted HR = 0.90, 95% CI = 0.65-1.23, *P* = 0.030 for heterogeneity test, Supplementary Table 4). Therefore, a gene-drink status interaction analysis was carried out, and a statistically significant multiplicative interaction was observed (multiplicative interaction analysis: *P* = 0.004 for rs11756089; *P* = 0.002 for rs2076173; *P* = 0.002 for the combined genotypes, Table 4, Figures 2 and 3). Comparing to drinkers with rs11756089 CC genotype, non-drinkers with rs11756089 CT/TT genotypes had a significantly decreased mortality risk (adjusted HR = 0.39, 95% CI = 0.24-0.65, *P* < 0.001, Table 4 and Figure 2A). Compared to drinkers with rs2076173 TT genotype, non-drinkers with rs2076173 TC/CC genotypes had a 52% significant risk reduction of death (adjusted HR = 0.48, 95% CI = 0.32-0.74, *P* = 0.001, Table 4 and Figure 2B). When combining the two SNPs, non-drinkers with 1-4 protective alleles had better survival with MST of 19.25 months (adjusted HR = 0.48, 95% CI = 0.32-0.74, *P* = 0.001, Table 4 and Figure 3) than drinkers with 0 protective allele.

**Table 4:** Interaction between variants genotypes and drinking status

Combined effects	SNP	Drinking status	Patients	Deaths	MST(months)	Adjusted HR (95% CI) ^a^	*P* ^a^
	rs11756089						
0	CC	Yes	167	137	12.06	1.00	
1	CT/TT	Yes	32	26	9.99	1.12 (0.73-1.72)	0.618
2	CC	No	90	72	16.69	0.79 (0.55-1.14)	0.210
3	CT/TT	No	36	22	22.67	0.39 (0.24-0.65)	<0.001
*P* for multiplicative interaction						0.004	
	rs2076173						
0	TT	Yes	95	80	12.85	1.00	
1	TC/CC	Yes	106	84	12.06	0.87 (0.64-1.19)	0.379
2	TT	No	47	40	15.93	0.93 (0.59-1.47)	0.765
3	TC/CC	No	78	54	19.25	0.48 (0.32-0.74)	0.001
*P* for multiplicative interaction						0.002	
Combined genotypes (rs11756089-T and rs2076173-C)							
0	0	Yes	93	78	11.30	1.00	
1	1-4	Yes	106	85	12.06	0.88 (0.64-1.20)	0.409
2	0	No	47	40	15.93	0.93 (0.59-1.48)	0.769
3	1-4	No	78	54	19.25	0.48 (0.32-0.74)	0.001
*P* for multiplicative interaction						0.002	

MST, median survival time; HR, hazard ratio; CI, confidence intervals.

^a^ Adjusted for age, gender, smoking and drinking status, BCLC stage, and chemotherapy or TACE status.

## DISCUSSION

In the present study, we investigated the associations of seven potentially functional *TEADs* SNPs with the clinical outcome in Chinese patients with intermediate or advanced HCC. The C allele of *TEAD3* rs2076173 and the T allele of *TEAD3* rs11756089 were identified as protective alleles. Patients who are carrying of at least one protective allele had significantly longer overall survival time than those without, especially for those non-drinkers. The prognostic value of these protective alleles was independent of age, gender, smoking and drinking status, BCLC stage, and chemotherapy or TACE status.

As transcriptional factors, TEADs have been found to be related with various human cancers by promoting cell proliferation and inhibiting apoptosis. The transcriptional function of TEADs was affected by three groups of coactivators including YAP/transcriptional coactivator with PDZ-binding motif (TAZ), vgll proteins, and p160 family of nuclear receptor coactivators [16]. TEAD1/2 enhanced *mesothelin* transcription which was frequently overexpressed in pancreatic and ovarian cancer [17, 18]. TEAD4 was found overexpressed in breast cancer to induce cell proliferation and tumor growth by inhibiting *p27* transcription [19]. TEAD1/4 overexpression was considered as prognostic marker for prostate cancer [20]. In human, TEAD3 was reported to regulate the transcription of 3 beta-hydroxysteroid dehydrogenase/isomerase (HSD3B), which was involved in the degradation of androstenone in the liver [21, 22]. Silencing of TEAD3 showed abrogation of radiation-induced adaptive response in mice, suggesting that TEAD3 may play a role in radiation-induced apoptosis, proliferation and differentiation [23]. Association of TEAD3 and Hippo-YAP signaling pathway was found in renal clear carcinoma [24], ovarian cancer [4] and hepatocellular carcinoma [9].

Here, we found Chinese patients who were carrying rs2076173 TC/CC or rs11756089 CT/TT genotypes of *TEAD3*, appeared to have a prolonged overall survival. The SNP rs2076173 (T>C) was located at the 3’-untranslated region of TEAD3. According to the web-based SNP analysis tool (SNPinfo: https://snpinfo.niehs.nih.gov/snpinfo/snpfunc.html), rs2076173 may be located at miRNAs (has-miR-455-3p and hs-miR-766)-binding site, which was likely to disrupt miRNA-target interaction and result in the deregulation of target gene expression. As no different binding energy changed in the two A/G alleles for the hsa-miR-455-3p, and binding energy for the hs-miR-766 was available only in the A allele in the SNPinfo database, further *in vitro* experiments, such as luciferase assay, were needed to validate our polymorphic functional hypothesis. Moreover, it was associated with the expression of *DEP6* (Effect size = -0.24, *P* = 0.0000062), *TEAD3* (Effect size = -0.32, *P* = 0.000011) and *RPL10A* (Effect size = 0.34, *P* = 0.000021) according to the database of GTExPortal (http://www.gtexportal.org). The SNP rs11756089 (C>T), a synonymous variant, may be served as exonic splicing enhancer (ESE) or exonic splicing silencer (ESS) according to the SNPinfo analysis tool. It was also associated with the expression of *TEAD3* (Effect size = -0.40, *P* = 0.000012) according to the database of GTExPortal. These evidences for the SNPs seem to be biologically plausible, but further functional analyses of the regions including the SNPs are needed.

HCC is a severe and complex disease caused by a combination of genetic and environmental factors [25, 26]. Previous studies showed that gene-gene and gene-environment interactions were implicated in HCC development [27, 28]. In this study, we identified the C allele of rs2076173 and the T allele of rs11756089 were protective alleles for HCC patients, especially for those nondrinkers. Patients who are carrying an increased number of these protective alleles will give a better survival outcome. In this cohort, patients who had 3-4 protective alleles had a significantly prolonged survival time of 19.25 months. For drinkers with none of these allele, the median survival time was 11.3 months, whereas, the MST was 19.25 months for those nondrinkers with 1-4 protective alleles. This finding suggested that the two SNPs may be involved in the process of gene-environment interactions.

To our knowledge, this is the first study to investigate the association between TEADs SNPs with HCC survival. Among the 7 SNPs, rs2076173 and rs11756089 polymorphisms of *TEAD3* were identified to be significantly associated with HCC patients. There are several limitations in our study. The results are based on a relatively small cohort of only 331 Chinese HBV-related HCC patients and our study was lack of replication cohort. Further validation will be needed in a larger cohort. Meanwhile, the functions of rs2076173 and rs11756089 have not been fully characterized. Related investigation is needed to further reveal the functions of this polymorphism on TEAD3 and their potential targets. In summary, our results demonstrated the potential use of *TEAD3* polymorphisms as prognostic markers for intermediate and advanced HCC patients. These data may provide a basis for rational HCC surveillance, therapeutics strategies development and medicinal individualization of HCC patients.

## MATERIALS AND METHODS

### Patients and samples collection

A total of 331 HCC patients of Barcelona Clinic Liver Cancer (BCLC) staging system stage B or C were recruited from Nantong Tumor Hospital (Nantong, People’s Republic of China) and Nanjing First Hospital, Nanjing Medical University (Nanjing, People’s Republic of China) from January 2006 to December 2010. Patients were diagnosed with HCC based on histopathological examination, or the measurement of serological α-fetoprotein level (>400 ng/mL) and imaging examination by magnetic resonance imaging and/or computerized tomography, as described previously [29]. Patients were followed up every 3 months from the date of enrollment until death or to the last follow-up date (January 14, 2013). Individuals who smoked 1 cigarette per day for over 1 year were defined as smokers, and those who consumed one or more alcohol drinks a week for over 6 months were categorized as alcohol drinkers. All of them were serologically confirmed hepatitis B virus (HBV) positive without receiving any surgical treatment. The study was approved by the institutional review board of Nanjing Medical University (Nanjing, People’s Republic of China). Signed informed consents were collected from enrolled patients for the use of clinical specimens in medical research.

### Serological tests

HBsAg, anti-HBs, anti-HBc and anti-HCV were detected by the enzyme-linked immunosorbent assay (Kehua Bio-engineering Co., Ltd., Shanghai, China) following the manufacturer’s instructions as described previously [30].

### SNPs selection and genotyping

All common (minor allele frequency, MAF > 0.05 in Chinese or Asians) polymorphisms in *TEAD1, TEAD2, TEAD3* and *TEAD4* of potentially functional, that is, located at 5’-flanking regions (5’-FRs), 5’-untranslated regions (5’-UTRs), coding regions, or 3’-UTRs according to NCBI dbSNPs (last search date: November 2014) were identified. SNPs that were demonstrated to be of biological significance or associated with gene expression and/or cancer risk/survival according to the literature review were also included. If SNPs are in high linkage disequilibrium (LD) (r^2^ > 0.8), only one SNP were genotyped. As a result, seven SNPs (*TEAD1* rs2304733 and rs10831923, *TEAD2* rs12104362 and rs3745305, *TEAD3* rs11756089 and rs2076173, *TEAD4* rs7135838) were selected for genotyping. Genomic DNA was extracted from leukocyte pellets by traditional proteinase K digestion, phenol-chloroform extraction and ethanol precipitation. All SNPs were genotyped using the Sequenom MassARRAY iPLEX platform (Sequenom Inc). The primers for the seven SNPs are shown in Supplementary Table 1. More than 5% of the samples were randomly selected for repeated genotyping, yielding a 100% concordance. The success rates of genotyping for the seven SNPs were all above 95%.

### Statistical analysis

Survival time was calculated from the date of HCC diagnosis to the date of death of any cause or last follow-up. Hardy-Weinberg equilibrium was assessed within patients by using a goodness-of-fit χ^2^ test. Mean survival time was presented when the median overall survival time (MST) could not be calculated. Kaplan-Meier method and log-rank test were performed to compare the survival time in different subgroups categorized by patient characteristics, clinical features and genotypes. Univariate and multivariable Cox proportional hazard regression analyses were performed to estimate the crude or adjusted HR and their 95% CIs, with adjustment of age, gender, smoking status, drinking status, BCLC stage, and chemotherapy or transcatheter hepatic arterial chemoembolization (TACE) status. Cox stepwise regression model was conducted to determine predictive factors for HCC prognosis, with a significance level of 0.050 for entering and 0.051 for removing the respective explanatory variables. The Chi-square-based *Q* test was applied to test the heterogeneity of associations between subgroups. Analyses were carried out using Statistical Analysis System software (version 9.1.3; SAS Institute, Cary, NC, USA). All tests were two-sided and the criterion of statistical significance was set at *P* < 0.05.

## SUPPLEMENTARY MATERIALS TABLES



## References

[1] Chen W, Zheng R, Baade PD, Zhang S, Zeng H, Bray F, Jemal A, Yu XQ, He J (2016). Cancer statistics in China, 2015. CA Cancer J Clin.

[2] Perra A, Kowalik MA, Ghiso E, Ledda-Columbano GM, Di Tommaso L, Angioni MM, Raschioni C, Testore E, Roncalli M, Giordano S, Columbano A (2014). YAP activation is an early event and a potential therapeutic target in liver cancer development. J Hepatol.

[3] Qiao Y, Lin SJ, Chen Y, Voon DC, Zhu F, Chuang LS, Wang T, Tan P, Lee SC, Yeoh KG, Sudol M, Ito Y (2016). RUNX3 is a novel negative regulator of oncogenic TEAD-YAP complex in gastric cancer. Oncogene.

[4] Xia Y, Zhang YL, Yu C, Chang T, Fan HY (2014). YAP/TEAD co-activator regulated pluripotency and chemoresistance in ovarian cancer initiated cells. PLoS One.

[5] Zhou Y, Huang T, Cheng AS, Yu J, Kang W, To KF (2016). The TEAD family and its oncogenic role in promoting tumorigenesis. Int J Mol Sci.

[6] Lai D, Ho KC, Hao Y, Yang X (2011). Taxol resistance in breast cancer cells is mediated by the hippo pathway component TAZ and its downstream transcriptional targets Cyr61 and CTGF. Cancer Res.

[7] Xu MZ, Chan SW, Liu AM, Wong KF, Fan ST, Chen J, Poon RT, Zender L, Lowe SW, Hong W, Luk JM (2011). AXL receptor kinase is a mediator of YAP-dependent oncogenic functions in hepatocellular carcinoma. Oncogene.

[8] Mizuno T, Murakami H, Fujii M, Ishiguro F, Tanaka I, Kondo Y, Akatsuka S, Toyokuni S, Yokoi K, Osada H, Sekido Y (2012). YAP induces malignant mesothelioma cell proliferation by upregulating transcription of cell cycle-promoting genes. Oncogene.

[9] Zhao B, Ye X, Yu J, Li L, Li W, Li S, Yu J, Lin JD, Wang CY, Chinnaiyan AM, Lai ZC, Guan KL (2008). TEAD mediates YAP-dependent gene induction and growth control. Genes Dev.

[10] Yimlamai D, Christodoulou C, Galli GG, Yanger K, Pepe-Mooney B, Gurung B, Shrestha K, Cahan P, Stanger BZ, Camargo FD (2014). Hippo pathway activity influences liver cell fate. Cell.

[11] Zhao B, Kim J, Ye X, Lai ZC, Guan KL (2009). Both TEAD-binding and WW domains are required for the growth stimulation and oncogenic transformation activity of yes-associated protein. Cancer Res.

[12] Liu-Chittenden Y, Huang B, Shim JS, Chen Q, Lee SJ, Anders RA, Liu JO, Pan D (2012). Genetic and pharmacological disruption of the TEAD-YAP complex suppresses the oncogenic activity of YAP. Genes Dev.

[13] Yuan H, Liu H, Liu Z, Zhu D, Amos CI, Fang S, Lee JE, Wei Q (2015). Genetic variants in Hippo pathway genes YAP1, TEAD1 and TEAD4 are associated with melanoma-specific survival. Int J Cancer.

[14] Qi Y, Yu J, Han W, Fan X, Qian H, Wei H, Tsai YH, Zhao J, Zhang W, Liu Q, Meng S, Wang Y, Wang Z (2016). A splicing isoform of TEAD4 attenuates the Hippo-YAP signalling to inhibit tumour proliferation. Nat Commun.

[15] Xie K, Liu J, Zhu L, Liu Y, Pan Y, Wen J, Ma H, Zhai X, Hu Z (2013). A potentially functional polymorphism in the promoter region of let-7 family is associated with survival of hepatocellular carcinoma. Cancer Epidemiol.

[16] Pobbati AV, Hong W (2013). Emerging roles of TEAD transcription factors and its coactivators in cancers. Cancer Biol Ther.

[17] Hucl T, Brody JR, Gallmeier E, Iacobuzio-Donahue CA, Farrance IK, Kern SE (2007). High cancer-specific expression of mesothelin (MSLN) is attributable to an upstream enhancer containing a transcription enhancer factor dependent MCAT motif. Cancer Res.

[18] Frierson HF, Moskaluk CA, Powell SM, Zhang H, Cerilli LA, Stoler MH, Cathro H, Hampton GM (2003). Large-scale molecular and tissue microarray analysis of mesothelin expression in common human carcinomas. Hum Pathol.

[19] Han W, Jung EM, Cho J, Lee JW, Hwang KT, Yang SJ, Kang JJ, Bae JY, Jeon YK, Park IA, Nicolau M, Jeffrey SS, Noh DY (2008). DNA copy number alterations and expression of relevant genes in triple-negative breast cancer. Genes Chromosomes Cancer.

[20] Knight JF, Shepherd CJ, Rizzo S, Brewer D, Jhavar S, Dodson AR, Cooper CS, Eeles R, Falconer A, Kovacs G, Garrett MD, Norman AR, Shipley J (2008). TEAD1 and c-Cbl are novel prostate basal cell markers that correlate with poor clinical outcome in prostate cancer. Br J Cancer.

[21] Peng L, Huang Y, Jin F, Jiang SW, Payne AH (2004). Transcription enhancer factor-5 and a GATA-like protein determine placental-specific expression of the Type I human 3beta-hydroxysteroid dehydrogenase gene, HSD3B1. Mol Endocrinol.

[22] Payne AH, Abbaszade IG, Clarke TR, Bain PA, Park CH (1997). The multiple murine 3 beta-hydroxysteroid dehydrogenase isoforms: structure, function, and tissue- and developmentally specific expression. Steroids.

[23] Vares G, Wang B, Tanaka K, Shang Y, Taki K, Nakajima T, Nenoi M (2011). Gene silencing of Tead3 abrogates radiation-induced adaptive response in cultured mouse limb bud cells. J Radiat Res.

[24] Matsuura K, Nakada C, Mashio M, Narimatsu T, Yoshimoto T, Tanigawa M, Tsukamoto Y, Hijiya N, Takeuchi I, Nomura T, Sato F, Mimata H, Seto M (2011). Downregulation of SAV1 plays a role in pathogenesis of high-grade clear cell renal cell carcinoma. BMC Cancer.

[25] Farazi PA, DePinho RA (2006). Hepatocellular carcinoma pathogenesis: from genes to environment. Nat Rev Cancer.

[26] Tang R, Liu H, Yuan Y, Xie K, Xu P, Liu X, Wen J (2017). Genetic factors associated with risk of metabolic syndrome and hepatocellular carcinoma. Oncotarget.

[27] Chen CJ, Chen DS (2002). Interaction of hepatitis B virus, chemical carcinogen, and genetic susceptibility: multistage hepatocarcinogenesis with multifactorial etiology. Hepatology.

[28] Yu MW, Yang SY, Chiu YH, Chiang YC, Liaw YF, Chen CJ (1999). A p53 genetic polymorphism as a modulator of hepatocellular carcinoma risk in relation to chronic liver disease, familial tendency, and cigarette smoking in hepatitis B carriers. Hepatology.

[29] Shen L, Wen J, Zhao T, Hu Z, Song C, Gu D, He M, Lee NP, Xu Z, Chen J (2016). A genetic variant in large tumor suppressor kinase 2 of Hippo signaling pathway contributes to prognosis of hepatocellular carcinoma. Onco Targets Ther.

[30] Hu L, Zhai X, Liu J, Chu M, Pan S, Jiang J, Zhang Y, Wang H, Chen J, Shen H, Hu Z (2012). Genetic variants in human leukocyte antigen/DP-DQ influence both hepatitis B virus clearance and hepatocellular carcinoma development. Hepatology.

