# Supplementary material for: The prognostic impacts of TEA domain (TEAD) transcription factor polymorphisms in Chinese hepatocellular carcinoma patients
Source: Oncotarget. 2017 Jul 17;8(41):69823–32. doi: 10.18632/oncotarget.19310 (PMC5642519; doi:10.18632/oncotarget.19310)
Supplement: Supplementary file 1 [file oncotarget-08-69823-s001.pdf]

The prognostic impacts of *TEA domain (TEAD)* transcription factor polymorphisms in Chinese hepatocellular carcinoma patients

SUPPLEMENTARY MATERIALS

Supplementary Table 1: Information of primers and probes for Sequenom allelic discrimination

**Polymorphism**		**Sequence (5'-3')**
rs2304733	2nd-PCR Primer	ACGTTGGATGGTAGGCCTGCTGCACAAAAG
	1st-PCR Primer	ACGTTGGATGTGCAGATGCGATCTCTTAAC
	Extend Primer	TGCGGCACAAAAGGCTTGAC
rs10831923	2nd-PCR Primer	ACGTTGGATGCACTGAAGTTCTAGGACAGC
	1st-PCR Primer	ACGTTGGATGTCCTTAAGGGAAGCCCCATC
	Extend Primer	GCTCGCCTGGTCATA
rs12104362	2nd-PCR Primer	ACGTTGGATGTGAGTCTGAGGGAGGAGAAG
	1st-PCR Primer	ACGTTGGATGACTCTGAGAACACTCCCATC
	Extend Primer	AGAAGGAGCTGGATTTCT
rs3745305	2nd-PCR Primer	ACGTTGGATGTTGCTTATGGTCGCACAGAG
	1st-PCR Primer	ACGTTGGATGAGCTACGTGGAAGCCAGACT
	Extend Primer	CCAGTGCGTGATGGA
rs11756089	2nd-PCR Primer	ACGTTGGATGATAAACCGTCTCTCTACCTG
	1st-PCR Primer	ACGTTGGATGGGCCGAAATGAGTTGATTGC
	Extend Primer	AACGTCTCTCTACCTGTTTTCT
rs2076173	2nd-PCR Primer	ACGTTGGATGCAAAGGGGCAAGGAAGTGTG
	1st-PCR Primer	ACGTTGGATGAAGGGAGTCAGTGAGCAGAG
	Extend Primer	CCTCAGTCCAACCCC
rs7135838	2nd-PCR Primer	ACGTTGGATGTGGACACTCGGGACTTTGG
	1st-PCR Primer	ACGTTGGATGGTGCTGGGTCTCTTCTGTTG
	Extend Primer	CCGGTAGAGGCAGAACCGAGA

Supplementary Table 2: Stratification analysis of rs11756089 genotypes associated with HCC patients' survival

Variables	rs11756089 (patients / deaths)			
	**CC**	**CT/TT**	**Adjusted HR (95% CI) [a]**	***P* for heterogeneity**
Age				
<=53	135/112	35/25	0.74 (0.46-1.18)	0.836
>53	122/97	33/23	0.69 (0.43-1.09)	
Gender				
Male	223/181	55/39	0.42 (0.18-0.94)	0.139
Female	34/28	13/10	0.83 (0.58-1.20)	
Smoking status				
No	89/71	30/19	0.51 (0.30-0.87)	0.106
Yes	168/138	38/29	0.89 (0.59-1.35)	
Drinking status				
No	90/72	36/22	0.43 (0.27-0.71)	0.002
Yes	167/137	32/26	1.22 (0.79-1.90)	
BCLC stage				
Stage B	235/190	63/45	0.73 (0.52-1.03)	0.276
Stage C	22/19	5/3	0.31 (0.07-1.41)	
Chemotherapy or TACE				
None	76/67	15/10	0.57 (0.28-1.13)	0.383
Yes	181/142	53/38	0.81 (0.56-1.17)	

HCC: hepatocellular carcinoma; BCLC: Barcelona Clinic Liver Cancer stage; HR: hazard ratio; CI: confidence intervals; TACE: transcatheter hepatic arterial chemoembolization.

[a] Adjusted for age, gender, smoking and drinking status, BCLC stage, and chemotherapy or TACE status except for the stratification factor.

Supplementary Table 3: Stratification analysis of rs2076173 genotypes associated with HCC patients' survival

Variables	rs2076173 (patients / deaths)			
	TT	TC/CC	Adjusted HR (95% CI) [a]	*P* for heterogeneity
Age				
<=53	66/60	105/78	0.62 (0.44-0.89)	0.434
>53	76/60	79/60	0.76 (0.53-1.11)	
Gender				
Male	17/14	30/23	0.86 (0.41-1.78)	0.782
Female	125/106	154/115	0.77 (0.59-1.01)	
Smoking status				
No	45/38	74/52	0.58 (0.38-0.89)	0.154
Yes	97/82	110/86	0.85 (0.62-1.15)	
Drinking status				
No	47/40	78/54	0.50 (0.33-0.77)	0.031
Yes	95/80	106/84	0.89 (0.65-1.21)	
BCLC stage				
Stage B	132/111	167/125	0.71 (0.55-0.93)	0.562
Stage C	10/9	17/13	0.52 (0.19-1.46)	
Chemotherapy or TACE				
None	41/37	50/40	0.75 (0.47-1.20)	0.720
Yes	101/83	134/98	0.83 (0.62-1.12)	

HCC: hepatocellular carcinoma; BCLC: Barcelona Clinic Liver Cancer stage; HR: hazard ratio; CI: confidence intervals; TACE: transcatheter hepatic arterial chemoembolization.

[a] Adjusted for age, gender, smoking and drinking status, BCLC stage, and chemotherapy or TACE status except for the stratification factor.

Supplementary Table 4: Stratification analysis of combined genotypes (rs11756089-T and rs2076173-C) associated with HCC patients' survival

Variables	Combined effects (patients / deaths)			
	**0**	**1-4**	**Adjusted HR (95% CI) [a]**	***P* for heterogeneity**
Age				
<=53	65/59	105/78	0.63 (0.44-0.89)	0.444
>53	75/59	79/61	0.77 (0.53-1.12)	
Gender				
Male	17/14	30/23	0.86 (0.41-1.78)	0.807
Female	123/104	154/116	0.78 (0.59-1.02)	
Smoking status				
No	45/38	74/52	0.58 (0.38-0.89)	0.156
Yes	95/80	110/87	0.85 (0.62-1.16)	
Drinking status				
No	47/40	78/54	0.50 (0.33-0.77)	0.030
Yes	93/78	106/85	0.90 (0.65-1.23)	
BCLC stage				
Stage B	130/109	167/126	0.72 (0.55-0.94)	0.545
Stage C	10/9	17/13	0.52 (0.19-1.46)	
Chemotherapy or TACE				
None	41/37	50/40	0.75 (0.47-1.20)	0.690
Yes	99/81	134/99	0.84 (0.62-1.13)	

HCC: hepatocellular carcinoma; BCLC: Barcelona Clinic Liver Cancer stage; HR: hazard ratio; CI: confidence intervals; TACE: transcatheter hepatic arterial chemoembolization.

[a] Adjusted for age, gender, smoking and drinking status, BCLC stage, and chemotherapy or TACE status except for the stratification factor.
